# Reevaluating ‘seriousness’ in genetic conditions: balancing clinical criteria and lived experiences

**DOI:** 10.1038/s41431-025-01829-6

**Published:** 2025-03-15

**Authors:** Shizuko Takahashi, Rie Iizuka, Tsutomu Sawai

**Affiliations:** 1 Centre for Biomedical Ethics, Yong Loo Lin School of Medicine, National University of Singapore, Singapore, Singapore; 2 Department of Biomedical Ethics, University of Tokyo, Graduate School of Medicine, Tokyo, Japan; 3 Department of Bioethics and Humanities, University of Washington, Seattle, WA USA; 4 Center for Collaborative Sciences, Office of Research and Academic-Government-Community Collaboration, Headquarters for Co-creative Future Sciences, Hiroshima University, Hiroshima, Japan; 5 Graduate School of Humanities and Social Sciences, Hiroshima University, Hiroshima, Japan; 6 Institute for the Advanced Study of Human Biology (ASHBi), Kyoto University, Kyoto, Japan; 7 Uehiro Division for Applied Ethics, Graduate School of Humanities and Social Sciences, Hiroshima University, Hiroshima, Japan

**Keywords:** Medical ethics, Cancer genetics, Risk factors

Kleiderman et al.’s recent review, “Unpacking the Notion of ‘Serious’ Genetic Conditions: Towards Implementation in Reproductive Decision-Making” [1], offers a valuable framework for understanding the multifaceted concept of seriousness in genetic conditions [1]. Their framework, which emphasizes the core dimensions and procedural elements of seriousness, provides valuable insights into the Japanese context. While many countries have expanded access to preimplantation genetic testing for monogenic disorders (PGT-M), Japan remains notably restrictive, approving only 17 conditions under the stringent criteria established by the Japanese Society of Obstetrics and Gynecology (JSOG) [2]. The most recent case, involving retinoblastoma, was initially rejected as “treatable” and only approved after six years with appeals [3, 4], reflecting ongoing debates over three decades of what qualifies as “serious [5].” These challenges reflect the procedural elements outlined by Kleiderman et al., particularly regarding procedural fairness and patient-centered impacts.

To explore how the core dimensions and procedural elements of seriousness interact in Japan, we conducted a survey during our “Discussion about PGT-M with the Stakeholders” workshop in Hiroshima on January 11, 2025. The workshop aimed to facilitate dialog among stakeholders, including patients, healthcare professionals, and ethicists, on the ethical and regulatory challenges of PGT-M. Of the 102 participants, 78 completed the survey (response rate: 76.5%).

The survey, conducted in two phases, assessed participants’ perceptions before and after patient testimonies. Initially, a clinical geneticist explained the medical criteria for PGT-M, treatment options, and available support resources, addressing core dimensions such as “medical criteria,” “access/availability of treatment,” and “access/availability of support and resources.” Subsequently, individuals with hereditary cancer—including patients with retinoblastoma and BRCA2 mutations—shared their lived experiences. This juxtaposition of medical information with personal narratives allowed participants to reflect on how “individual and familial lived experiences,” a crucial core dimension, influence perceptions of seriousness.

Before hearing patient testimonies, 66% of respondents indicated they would consider PGT-M if faced with hereditary cancer, citing a desire to prevent the inheritance of debilitating conditions(Supplementary 1). Additionally, 89% believed that patients should be informed about PGT-M at the time of diagnosis, underscoring the importance of access to information. However, these perspectives shifted notably after hearing patient stories. The proportion of participants who viewed PGT-M primarily as a public health tool aimed at reducing societal burden significantly decreased (*p* = 0.005), with an increased emphasis on individual reproductive autonomy (Supplementary 2). Favorable attitudes toward marriage, pregnancy, and family planning with PGT-M rose from 54% pre-testimony to 71% post-testimony (Supplementary 1). These shifts align with the “patient/family-centered impacts” element in Kleiderman et al.‘s framework, highlighting how firsthand accounts can recalibrate notions of seriousness. Other results and the survey questionnaire are as indicated in Supplementary 3.

The retinoblastoma case presented at the workshop illustrates the friction between core dimensions and procedural elements in Kleiderman et al.’s framework. Despite the patient’s firsthand experience of living with retinoblastoma and raising a child affected by the condition, her PGT-M request was repeatedly denied under the rationale that the disease was “treatable.” Approval was granted only after extensive advocacy and increased societal awareness. This case exemplifies how procedural fairness in assessing seriousness can be compromised when lived experiences are undervalued relative to rigid medical criteria.

Respondents also highlighted barriers consistent with the procedural elements identified by Kleiderman et al. Many participants expressed concerns about opaque eligibility criteria and limited shared decision-making among patients, healthcare providers, and regulatory bodies. The absence of standardized protocols for discussing PGT-M at diagnosis was seen as a significant gap from the West, reflecting challenges in ensuring equitable access to information. Although the clinical criteria are explicit, the process of determining what qualifies as “serious” often lacks transparency, contributing to feelings of exclusion among patients navigating Japan’s healthcare system.

Historical and cultural contexts further complicate how seriousness is interpreted in Japan. The country’s eugenic history has fostered caution in the adoption of reproductive technologies like PGT-M [6]. Disability advocacy groups have raised concerns that expanding PGT-M access could perpetuate ableist attitudes. While such concerns are valid, they can sometimes overshadow patient autonomy, as our survey responses suggest. Balancing disability rights with reproductive choice remains a nuanced challenge, exemplifying the complexity of procedural elements in assessing seriousness.

Applying Kleiderman et al.’s framework to our survey data reveals substantial tension between the core dimensions and procedural elements in Japan’s approach to PGT-M. Medical criteria and societal considerations often dominate policy discussions, whereas individual and familial lived experiences—though central to patient well-being—are frequently marginalized in decision-making processes. The changes in participant perceptions following patient testimonies underscore the importance of incorporating diverse experiences into the evaluation of seriousness.

Given the significant emotional, ethical, and financial burdens hereditary cancer patients face, the complexities of assessing seriousness in PGT-M decisions become apparent when viewed through Kleiderman et al.‘s framework. The core dimensions—medical criteria, access to treatment and support, and individual and familial lived experiences—often clash with procedural elements like fairness in decision-making and patient-centered impacts. Our survey data reveal how patients’ lived experiences, a central dimension of seriousness, are frequently undervalued in Japan’s current regulatory process, while rigid adherence to medical criteria predominates.

As disability advocacy groups in the US have emphasized, reforms must also recognize diverse disability experiences within an ableist society [7], reflecting the procedural element of ensuring fairness in assessing seriousness. An inclusive policy framework, consistent with Kleiderman et al.‘s emphasis on balancing core dimensions and procedural elements, should accommodate multiple perspectives and individual choices while proactively addressing disability stigma. Integrating these considerations not only enhances reproductive autonomy but also fosters disability justice in genetics.

## Supplementary information

Supplementary Material(s)

## Data Availability

The data generated and analyzed during this study are available within the published article and its supplementary files. Additional data are available from the corresponding author upon reasonable request.
