# Supplementary material for: Reevaluating ‘seriousness’ in genetic conditions: balancing clinical criteria and lived experiences
Source: Eur J Hum Genet. 2025 Mar 15;33(6):699–700. doi: 10.1038/s41431-025-01829-6 (PMC12185707; doi:10.1038/s41431-025-01829-6)
Supplement: Supplementary file 1 — Supplementary Material(s) [file 41431_2025_1829_MOESM1_ESM.docx]

**Supplementary 1. Figure 1.** Willingness to Undergo PGT-M and Opinions about PGT-M. The Percentage of those Strongly Agee and Agree are shown. The Willingness to undergo PGT-M themselves was only asked pre-testimony.

**Supplementary 2. Figure 2. Changes in motivations to undergo PGT-M before and after patient testimony. Those who Strongly Agree-Agree are shown.**

**Supplementary 3. Table 1. Questionnaire Responses and Demographic Information of the Participants**. The survey was developed and hosted on Qualtrics. Of the 102 attendees, 78 completed the survey. Responses are shown in percentages(%). Highlighted in green are same questions asked before and after patient testimonies to examine the effect on participant’s perception on PGT-M.

| **Pre-PGT M Patient Presentation** | | | | | | | | | | | | | | | | | | | | | | | | | | | | | | | | | | | | | | | | | | | | | | | | | | | | |
| --- | --- | --- | --- | --- | --- | --- | --- | --- | --- | --- | --- | --- | --- | --- | --- | --- | --- | --- | --- | --- | --- | --- | --- | --- | --- | --- | --- | --- | --- | --- | --- | --- | --- | --- | --- | --- | --- | --- | --- | --- | --- | --- | --- | --- | --- | --- | --- | --- | --- | --- | --- | --- |
|  | **Question** | | | | | | | **Response (%)** | | | | | | | | | | | | | | | | | | | | | | | | | | | | | | | | | | | | | | | | | | | |  |
| Q1 | Would you like to undergo PGT-M if you had hereditary cancer? | | | | | | | Strongly agree | | | | | | | Agree | | | | | | | | | Indifferent | | | | | | | | | | | | | | | Slightly Disagree | | | | | | | | | | | Strongly Disagree | |  |
|  |  |  |  |  |  |  |  | 43 | | | | | | | 23 | | | | | | | | | 15 | | | | | | | | | | | | | | | 11 | | | | | | | | | | | 3 | |  |
| Q2 | What (will) made(make) you think of PGT-M? | | | | | | | I have hereditary cancer 22% | | | | | | | | | | | | | | | | | | | | | | | | | | | | | | | | | | | | | | | | | | | |  |
|  |  |  |  |  |  |  |  | My family has hereditary cancer 6% | | | | | | | | | | | | | | | | | | | | | | | | | | | | | | | | | | | | | | | | | | | |  |
|  |  |  |  |  |  |  |  | Concern for passing hereditary condition24% | | | | | | | | | | | | | | | | | | | | | | | | | | | | | | | | | | | | | | | | | | | |  |
|  |  |  |  |  |  |  |  | Other Concerns 49% | | | | | | | | | | | | | | | | | | | | | | | | | | | | | | | | | | | | | | | | | | | |  |
| Q3 | Do you think it is selfish to undergo PGT-M? | | | | | | | Strongly  Agree | | | | | | | | | | Indifferent | | | | | | | | | | | | | Slightly do not think so. | | | | | | | | | | | | | | | Not at all | | | | | |  |
|  |  |  |  |  |  |  |  | 4 | | | | | | | | | | 7 | | | | | | | | | | | | | 24 | | | | | | | | | | | | | | | 65 | | | | | |  |
| Q4 | Are carriers responsible to undergo PGT-M? | | | | | | | Strongly Agree | | | | | | | | Agree | | | | | | | | | Indifferent | | | | | | | | | | | | | Slightly Disagree | | | | | | | | | | | Strongly Disagree | | |  |
|  |  |  |  |  |  |  |  | 2 | | | | | | | | 2 | | | | | | | | | 29 | | | | | | | | | | | | | 25 | | | | | | | | | | | 40 | | |  |
| Q5 | What is your rationale for Q4? (multiple selection) | | | | | | | Improving Health of the future Child | | | | | | | | | | | | | | | | | | | | | | | | 71 | | | | | | | | | | | | | | | | | | | |  |
|  |  |  |  |  |  |  |  | Reducing the Psychological and Economical Burden for the Family | | | | | | | | | | | | | | | | | | | | | | | | 33 | | | | | | | | | | | | | | | | | | | |  |
|  |  |  |  |  |  |  |  | Reducing the effect of hereditary cancers in society | | | | | | | | | | | | | | | | | | | | | | | | 21 | | | | | | | | | | | | | | | | | | | |  |
|  |  |  |  |  |  |  |  | Not having the child experience the same trauma | | | | | | | | | | | | | | | | | | | | | | | | 33 | | | | | | | | | | | | | | | | | | | |  |
|  |  |  |  |  |  |  |  | Others | | | | | | | | | | | | | | | | | | | | | | | | 29 | | | | | | | | | | | | | | | | | | | |  |
| Q6 |  | | | | | | | Strongly Agree | | | | | Agree | | | | | | | | | | Indifferent | | | | | | | | | | | | | | | | | Slightly Disagree | | | | | | | | | | | Strongly Disagree |  |
|  | 1. Do you think it is limiting to have PGT-M? | | | | | | | 9 | | | | | 6 | | | | | | | | | | 9 | | | | | | | | | | | | | | | | | 42 | | | | | | | | | | | 34 |  |
|  | 1. Do you think it is too restrictive that PGT-M is not available in Japan? | | | | | | | 40 | | | | | 47 | | | | | | | | | | 5.5 | | | | | | | | | | | | | | | | | 5.5 | | | | | | | | | | | 2 |  |
|  | 1. Do you think that patients can be more positive about marriage, pregnancy and have starting a family with PGT-M? | | | | | | | 19 | | | | | 45 | | | | | | | | | | 23 | | | | | | | | | | | | | | | | | 13 | | | | | | | | | | | 0 |  |
| Q7 | Which do you think is the most appropriate national health insurance coverage for PGT-M? | | | | | | | Both PGT-M and IVF(ICSI) | | | | | | | | | | | | | | | | | | | | | | | | 54.5 | | | | | | | | | | | | | | | | | | | |  |
|  |  |  |  |  |  |  |  | Just IVF (ICSI) | | | | | | | | | | | | | | | | | | | | | | | | 31 | | | | | | | | | | | | | | | | | | | |  |
|  |  |  |  |  |  |  |  | All out of pocket | | | | | | | | | | | | | | | | | | | | | | | | 14.5 | | | | | | | | | | | | | | | | | | | |  |
| **Post-PGT M Patient Presentation** | | | | | | | | | | | | | | | | | | | | | | | | | | | | | | | | | | | | | | | | | | | | | | | | | | | | |
| Q1 | What would be the problems you would face with PGT-M? (multiple answer) | | | | | | | Problems with “selecting life.” | | | | | | | | | | | | | | | | | | | | | | | | 48 | | | | | | | | | | | | | | | | | | | |  |
|  |  |  |  |  |  |  |  | Problems with the child’s “right not to know.” | | | | | | | | | | | | | | | | | | | | | | | | 30 | | | | | | | | | | | | | | | | | | | |  |
|  |  |  |  |  |  |  |  | Pressures from society and culture. | | | | | | | | | | | | | | | | | | | | | | | | 44 | | | | | | | | | | | | | | | | | | | |  |
|  |  |  |  |  |  |  |  | Economic burden of PGT-M. | | | | | | | | | | | | | | | | | | | | | | | | 72 | | | | | | | | | | | | | | | | | | | |  |
|  |  |  |  |  |  |  |  | Other (Free Text) | | | | | | | | | | | | | | | | | | | | | | | | 9 | | | | | | | | | | | | | | | | | | | |  |
| Q2 | What kind of impact would availability of PGT-M have on society? (free text) | | | | | | | 39 people responded with free text. | | | | | | | | | | | | | | | | | | | | | | | | | | | | | | | | | | | | | | | | | | | |  |
| Q3 | Do you think hereditary cancer patients need to be informed of PGT-M? | | | | | | | Strongly Agree | | | | | | | | | Agree | | | | | | | | | | | | | Slightly Disagree | | | | | | | | | | | | | Strongly Disagree | | | | | | | | |  |
|  |  |  |  |  |  |  |  | 62 | | | | | | | | | 27 | | | | | | | | | | | | | 9 | | | | | | | | | | | | | 2 | | | | | | | | |  |
| Q4 | When should a patient be informed of PGT-M? (multiple choice) | | | | | | | When tested positive for the gene. | | | | | | | | | | | | | | | | | | | | | | 51 | | | | | | | | | | | | | | | | | | | | | |  |
|  |  |  |  |  |  |  |  | Along with fertility preservation information prior to cancer treatment. | | | | | | | | | | | | | | | | | | | | | | 45 | | | | | | | | | | | | | | | | | | | | | |  |
|  |  |  |  |  |  |  |  | When thinking about family planning. | | | | | | | | | | | | | | | | | | | | | | 44 | | | | | | | | | | | | | | | | | | | | | |  |
|  |  |  |  |  |  |  |  | As a part of sexual health education. | | | | | | | | | | | | | | | | | | | | | | 40 | | | | | | | | | | | | | | | | | | | | | |  |
|  |  |  |  |  |  |  |  | Other | | | | | | | | | | | | | | | | | | | | | | 11 | | | | | | | | | | | | | | | | | | | | | |  |
| Q5 | Who should inform the patient? | | | | | | | Physicians | | | | | | | | | | | | | | | | | | | | | | 56 | | | | | | | | | | | | | | | | | | | | | |  |
|  |  |  |  |  |  |  |  | Genetic Counsellors | | | | | | | | | | | | | | | | | | | | | | 87 | | | | | | | | | | | | | | | | | | | | | |  |
|  |  |  |  |  |  |  |  | Peers with Hereditary Cancer | | | | | | | | | | | | | | | | | | | | | | 11 | | | | | | | | | | | | | | | | | | | | | |  |
|  |  |  |  |  |  |  |  | Family | | | | | | | | | | | | | | | | | | | | | | 5 | | | | | | | | | | | | | | | | | | | | | |  |
|  |  |  |  |  |  |  |  | Other | | | | | | | | | | | | | | | | | | | | | | 11 | | | | | | | | | | | | | | | | | | | | | |  |
| Q6 | Are carriers responsible to undergo PGT-M? | | | | | | | Strongly Agree | | | | | Agree | | | | | | | | | | | | | Indifferent | | | | | | | | | | Slightly Disagree | | | | | | | | | | | Strongly Disagree | | | | |  |
|  |  |  |  |  |  |  |  | 0 | | | | | 0 | | | | | | | | | | | | | 40 | | | | | | | | | | 20 | | | | | | | | | | | 40 | | | | |  |
| Q7 | What is your rationale for Q6? (multiple selection) | | | | | | | Improving Health of the future Child | | | | | | | | | | | | | | | | | | | | | 62 | | | | | | | | | | | | | | | | | | | | | | |  |
|  |  |  |  |  |  |  |  | Reducing the Psychological and Economical Burden for the Family | | | | | | | | | | | | | | | | | | | | | 38 | | | | | | | | | | | | | | | | | | | | | | |  |
|  |  |  |  |  |  |  |  | Reducing the effect of hereditary cancers in society | | | | | | | | | | | | | | | | | | | | | 6 | | | | | | | | | | | | | | | | | | | | | | |  |
|  |  |  |  |  |  |  |  | Not having the child experience the same trauma | | | | | | | | | | | | | | | | | | | | | 41 | | | | | | | | | | | | | | | | | | | | | | |  |
|  |  |  |  |  |  |  |  | Others | | | | | | | | | | | | | | | | | | | | | 26 | | | | | | | | | | | | | | | | | | | | | | |  |
| Q8 |  | | | | | Strongly Agree | | | | | Agree | | | | | | | | | | | Indifferent | | | | | | | | | | | | | Slightly Disagree | | | | | | | | | Strongly Disagree | | | | | | | |  |
|  | 1. Do you think it is limiting to have PGT-M? | | | | | 10 | | | | | 8 | | | | | | | | | | | 13 | | | | | | | | | | | | | 40 | | | | | | | | | 29 | | | | | | | |  |
|  | 1. Do you think it is too restrictive that PGT-M is not available in Japan? | | | | | 46 | | | | | 48 | | | | | | | | | | | 2 | | | | | | | | | | | | | 4 | | | | | | | | | 0 | | | | | | | |  |
|  | 1. Do you think that patients can be more positive about marriage, pregnancy and have starting a family with PGT-M? | | | | | 27 | | | | | 44 | | | | | | | | | | | 21 | | | | | | | | | | | | | 8 | | | | | | | | | 0 | | | | | | | |  |
| **Demographic Information (%)** | | | | | | | | | | | | | | | | | | | | | | | | | | | | | | | | | | | | | | | | | | | | | | | | | | | | |
| Age | 18-24 | | 25-34 | | | | | | 35-44 | | | | | | | | | | 45-54 | | | | | | | | | | | | | | 55-64 | | | | | | | | | 65- | | | | | | | | | |  |
|  | 4 | | 18 | | | | | | 18 | | | | | | | | | | 35 | | | | | | | | | | | | | | 16 | | | | | | | | | 9 | | | | | | | | | |  |
| Gender | Male | | | | | | | | Female | | | | | | | | | | | | | | | | | | | | | | | | Non-Binary | | | | | | | | | | | | | | | | | | |  |
|  | 24 | | | | | | | | 74 | | | | | | | | | | | | | | | | | | | | | | | | 2 | | | | | | | | | | | | | | | | | | |  |
| Child | Yes | | | | | | | | | | | | | | | | | | | | No | | | | | | | | | | | | | | | | | | | | | | | | | | | | | | |  |
|  | 60 | | | | | | | | | | | | | | | | | | | | 40 | | | | | | | | | | | | | | | | | | | | | | | | | | | | | | |  |
| Fertility Treatment | Yes | | | | | | | | | | | | | | | | | | | No | | | | | | | | | | | | | | | | | | | | | | | | | | | | | | | |  |
|  | 24 | | | | | | | | | | | | | | | | | | | 76 | | | | | | | | | | | | | | | | | | | | | | | | | | | | | | | |  |
| Have had Genetic Counselling | Yes | | | | | | | | | | | | | | | | | | | No | | | | | | | | | | | | | | | | | | | | | | | | | | | | | | | |  |
|  | 24 | | | | | | | | | | | | | | | | | | | 76 | | | | | | | | | | | | | | | | | | | | | | | | | | | | | | | |  |
| Marital Status | Married | | | | | | | | | Single | | | | | | | | | | | | | | | | | | | | | | | | Divorced/Living Separately | | | | | | | | | | | | | | | | | |  |
|  | 67 | | | | | | | | | 27 | | | | | | | | | | | | | | | | | | | | | | | | 5 | | | | | | | | | | | | | | | | | |  |
| Relation to Hereditary Cancer | Have hereditary cancer | | | | Family | | | | | | | Medical Professional | | | | | | | | | | | | | | | | Researcher (non-medical) | | | | | | | | | | | | | Other | | | | | | | | | | |  |
|  | 20 | | | | 4 | | | | | | | 56 | | | | | | | | | | | | | | | | 11 | | | | | | | | | | | | | 9 | | | | | | | | | | |  |
| Education | Middle School | | | | | | | | | | | | | | | | | | | 4 | | | | | | | | | | | | | | | | | | | | | | | | | | | | | | | |  |
|  | Completed Middle School | | | | | | | | | | | | | | | | | | | 5 | | | | | | | | | | | | | | | | | | | | | | | | | | | | | | | |  |
|  | Vocational School | | | | | | | | | | | | | | | | | | | 7 | | | | | | | | | | | | | | | | | | | | | | | | | | | | | | | |  |
|  | College | | | | | | | | | | | | | | | | | | | 3 | | | | | | | | | | | | | | | | | | | | | | | | | | | | | | | |  |
|  | Bachelor’s Degree | | | | | | | | | | | | | | | | | | | 23 | | | | | | | | | | | | | | | | | | | | | | | | | | | | | | | |  |
|  | Master’s Degree or equivalent (MA, MS, MBA, PhD, JD, MD, DDS) | | | | | | | | | | | | | | | | | | | 49 | | | | | | | | | | | | | | | | | | | | | | | | | | | | | | | |  |
|  | N/A | | | | | | | | | | | | | | | | | | | 4 | | | | | | | | | | | | | | | | | | | | | | | | | | | | | | | |  |
| Work | Full Time | | | Part Time | | | | | | Homemaker | | | | | | | | | | Student | | | | | | | | | | | | | | Retired | | | | | | | | | | | Other | | | | | | |  |
|  | 67 | | | 16 | | | | | | 4 | | | | | | | | | | 9 | | | | | | | | | | | | | | 2 | | | | | | | | | | | 2 | | | | | | |  |
| Annual Income  (1000yen) | 0-199 | 200-399 | | | | | 400-599 | | | | | | | 600-799 | | | | | | | | | | | | | 800-999 | | | | | | | | | | 1000- | | | | | | | | | | | N/A | | | | |
